# Analysis of a large dataset reveals haplotypes carrying putatively recessive lethal and semi-lethal alleles with pleiotropic effects on economically important traits in beef cattle

**DOI:** 10.1186/s12711-019-0452-z

**Published:** 2019-03-05

**Authors:** Janez Jenko, Matthew C. McClure, Daragh Matthews, Jennifer McClure, Martin Johnsson, Gregor Gorjanc, John M. Hickey

**Affiliations:** 1 The Roslin Institute and Royal (Dick) School of Veterinary Studies, The University of Edinburgh, Easter Bush, Midlothian, Scotland UK; 2 Irish Cattle Breeding Federation, Bandon, Co. Cork Ireland; 3 Department of Animal Breeding and Genetics, Swedish University of Agricultural Sciences, Box 7023, 750 07 Uppsala, Sweden

## Abstract

**Background:**

In livestock, deleterious recessive alleles can result in reduced economic performance of homozygous individuals in multiple ways, e.g. early embryonic death, death soon after birth, or semi-lethality with incomplete penetrance causing reduced viability. While death is an easy phenotype to score, reduced viability is not as easy to identify. However, it can sometimes be observed as reduced conception rates, longer calving intervals, or lower survival for live born animals.

**Methods:**

In this paper, we searched for haplotypes that carry putatively recessive lethal or semi-lethal alleles in 132,725 genotyped Irish beef cattle from five breeds: Aberdeen Angus, Charolais, Hereford, Limousin, and Simmental. We phased the genotypes in sliding windows along the genome and used five tests to identify haplotypes with absence of or reduced homozygosity. Then, we associated the identified haplotypes with 44,351 insemination records that indicated early embryonic death, and postnatal survival records. Finally, we assessed haplotype pleiotropy by estimating substitution effects on estimates of breeding value for 15 economically important traits in beef production.

**Results:**

We found support for one haplotype that carries a putatively recessive lethal (chromosome 16 in Simmental) and two haplotypes that carry semi-lethal alleles (chromosome 14 in Aberdeen Angus and chromosome 19 in Charolais), with population frequencies of 8.8, 15.2, and 14.4%, respectively. These three haplotypes showed pleiotropic effects on economically important traits for beef production. Their allele substitution effects are €2.30, €3.42, and €1.47 for the terminal index and €1.03, − €3.11, and − €0.88 for the replacement index, where the standard deviations for the terminal index are €22.52, €18.65, and €22.70 and for the replacement index they are €31.35, €29.82, and €35.79. We identified *ZFAT* as the candidate gene for semi-lethality in Aberdeen Angus, several candidate genes for the lethal Simmental haplotype, and no candidate genes for the semi-lethal Charolais haplotype.

**Conclusions:**

We analysed genotype, reproduction, survival, and production data to detect haplotypes that carry putatively recessive lethal or semi-lethal alleles in Irish beef cattle and identified one lethal and two semi-lethal haplotypes, which have pleiotropic effects on economically important traits in beef production.

**Electronic supplementary material:**

The online version of this article (10.1186/s12711-019-0452-z) contains supplementary material, which is available to authorized users.

## Background

Reproduction inefficiency has a major negative effect on profitability in cattle farming [1–4]. Over the last few decades, reproduction has deteriorated in both dairy and beef cattle in many countries, due to strong selection on production traits and their antagonistic relationship with reproduction [5, 6], and probably also to inbreeding. In recent years, this trend has begun to be readdressed in many countries by emphasising reproduction traits in animal recording and breeding goals [7, 8]. Furthermore, the advent of affordable high-density genotyping has enabled genomic selection jointly for production and reproduction traits [9, 10] and screening for recessive lethal alleles [11–15].

Recessive lethal alleles are one of the genetic causes of reproduction inefficiency, because they result in embryonic death when an embryo is homozygous. They can also reduce viability of embryos and liveborn animals when their effect is not fully expressed (incomplete penetrance). Death is an easy phenotype to score but reduced viability is not as easy to identify. However, in some cases, it is observed as reduced conception rates, longer calving intervals, or lower survival for live born animals. Such alleles tend to be rare, but if their frequency increases, the number of affected (homozygous) embryos increases and so does the economic loss. Recessive lethal alleles may increase in frequency in a population because of genetic drift, linkage with favourable alleles, and heterozygote advantage. For example, a recessive lethal allele shows heterozygote advantage if it has a positive pleiotropic effect on production traits thereby conferring an advantage to heterozygous carriers, but not to affected (homozygous) embryos [16]. This would lead to the selection of heterozygous carriers as parents of the next generation, propagate the recessive lethal allele across the population, and in turn significantly affect reproduction [17].

The availability of high-density genotype data has enabled screening of livestock populations for recessive lethal alleles. There are reported successes in dairy cattle [11–14], beef cattle [15], and pigs [18, 19]. The screening is based on the detection of the absence of homozygotes, either in whole populations or within carrier families. Several recessive haplotypes that show pleiotropic effects on yield, longevity and fertility in dairy cattle have been reported [20].

Recently, the Irish Cattle Breeding Federation (ICBF) has initiated large-scale genotyping of commercial beef cattle in Ireland [21]. To date, more than 1.3 million purebred and crossbred animals have been genotyped. These data will enable genomic selection to increase the productivity and reduce the environmental footprint of Irish cattle [22]. It can also be a powerful resource for biological discovery, including detection of recessive lethal or semi-lethal alleles, should they exist.

The objectives of our study were to discover haplotypes that carry putatively recessive lethal or semi-lethal alleles in the most numerous Irish beef cattle breeds, and to quantify their pleiotropic effects on traits under selection. We phased the genotypes in sliding windows along the genome for 132,725 purebred animals from five breeds and used five tests to identify haplotypes with absence or reduced homozygosity. We corroborated the identified haplotypes with reproduction and postnatal survival records. We found support for one haplotype that carries a putatively recessive lethal allele and two haplotypes that carry putatively recessive semi-lethal alleles. Substitution analysis showed that these haplotypes had pleiotropic effects on economically important traits in Irish beef cattle.

## Methods

In this paper, we analysed genotype, reproduction, survival, and production data to identify and validate haplotypes that carry putatively recessive lethal or semi-lethal alleles in Irish beef cattle. Briefly, our approach consisted of:

1. Genotype filtering, imputation, and phasing,
2. Identification of haplotypes that carry putatively recessive lethal or semi-lethal alleles,
3. Reproduction and survival analysis to corroborate the identified haplotypes,
4. Pleiotropy analysis to estimate haplotype effects on production traits,
5. Identification of candidate genes.

### Genotype filtering, imputation, and phasing

We used single nucleotide polymorphism (SNP) array data from the ICBF for purebred Aberdeen Angus, Charolais, Hereford, Limousin, and Simmental animals. The animals were genotyped with four different, but overlapping, Illumina SNP arrays (HD, IDBv1, IDBv2, and IDBv3; see Table 1), with most of the animals genotyped on the IDBv3 array (> 64%). We filtered the genotype data on autosomal chromosomes, missing information, and heterozygosity. We excluded SNPs with a low genotype call rate (< 0.90), and animals with a low genotype call rate (< 0.90) or high heterozygosity (> 6 standard deviations from mean heterozygosity). After filtering, 132,725 animals were available: 22,836 Aberdeen Angus; 39,472 Charolais; 12,678 Hereford; 45,965 Limousin; and 11,774 Simmental animals (Table 1). Finally, we reduced the SNPs to those that were present on the IDBv3 array and had a minor allele frequency higher than 0.05 within each breed. This decreased the number of SNPs to 40,146 for Aberdeen Angus; 41,366 for Charolais; 40,368 for Hereford; 40,951 for Limousin; and 39,907 for Simmental. We imputed missing genotypes in these reduced sets with AlphaImpute version 1.9.1 [23], and then they were phased with AlphaPhase version 1.9.1 [23] (using a core and tail length of 320 SNPs) into 20 SNP haplotypes in sliding windows. This means that the genotypes at each locus were phased 20 times in sliding windows, with the start of each window being moved along by one SNP at a time.

**Table 1:** Number of SNPs per array and number of genotyped animals after genotype filtering per breed and array

Number of genotyped animals per breed and array	Number of SNPs per array				Total
	HD	IDBv1	IDBv2	IDBv3	
	774,959	16,044	16,291	53,368	
Aberdeen Angus	532	1969	5409	14,926	22,836
Charolais	1,035	3950	10,868	23,619	39,472
Hereford	362	1759	2825	7,732	12,678
Limousin	1043	4134	10,717	30,071	45,965
Simmental	417	820	1798	8739	11,774
Total	3389	12632	31,617	85,087	132,725

### Identification of haplotypes that carry putatively recessive lethal or semi-lethal alleles

To identify haplotypes that carry putatively recessive lethal or semi-lethal alleles, the phased haplotypes from 132,725 animals were analysed for complete absence or a reduced level of homozygosity within the analysed breed (see Table 1). We used five different tests that were grouped into three categories: (i) population, (ii) carrier mating, and (iii) semi-lethality. The first and second categories are based on the probability of observing complete absence of recessive homozygous animals in the whole population or in the progeny from carrier matings. Thus, they assume that recessive lethal alleles are completely penetrant. The third category tests the null hypothesis that observed and expected numbers of recessive homozygous animals are equal. Thus, it is able to detect recessive semi-lethal alleles, with some homozygotes not suffering from deleterious effects or some haplotypes that imperfectly tag a recessive lethal allele. From this point on, we will use *HH* to denote wild type homozygotes, *Hh* to denote heterozygotes, and *hh* to denote homozygotes for a haplotype that carries putatively recessive lethal alleles.

#### Population test

The first test identifies haplotypes that carry putatively recessive lethal alleles based on their population frequency. The probability of observing no *hh* animals (*P*_*hh*_) depends on the *Hh* frequency (*c*) and the number of genotyped animals (*N*): *P*_*hh*_= (1 − *c*^2^/4)^*N*^. The number of expected *hh* animals (*E*_*hh*_) under these conditions is equal to: *E*_*hh*_= (*N*/4)·*c*^2^. This test is the same as that used by VanRaden et al. [12].

#### Carrier mating tests

The second category consists of two tests that identify haplotypes that carry putatively recessive lethal alleles based on the expected number of *hh* progeny from *Hh *× *Hh* matings. We analysed both sire × dam and sire × maternal grand-sire *Hh *× *Hh* matings. The probability of observing no *hh* animals follows a Bernoulli process and can be calculated as *P*_*hh*_= 0.75^*C*^ for *C* sire × dam *Hh *× *Hh* matings and *P*_*hh*_= 0.875^*C*^ for *C* sire × maternal grand-sire *Hh *× *Hh* matings. The expected number of *hh* progeny was calculated as *C*/4 for sire × dam *Hh *× *Hh* matings and *C*/8 for sire × maternal grand-sire *Hh *× *Hh* matings. These tests are partially the same as that used by VanRaden et al. [12].

#### Semi-lethality tests

The third category tests the null hypothesis that the observed and expected number of *hh* animals do not differ based on sire × dam *Hh *× *Hh* or sire × maternal grand-sire *Hh *× *Hh* matings. From the number of observed and expected *hh* (*O*_*hh*_ and *E*_*hh*_), *Hh* (*O*_*Hh*_ and *E*_*Hh*_), and *HH* (*O*_*HH*_ and *E*_*HH*_) progeny, we calculated a one-way Chi square test statistic: *χ*^2^ = (*O*_*hh*_ − *E*_*hh*_)^2^/*E*_*hh*_+ (*O*_*HH*+*Hh*_ − *E*_*HH*+*Hh*_)^2^/*E*_*HH*+*Hh*_. The test statistic has one degree of freedom.

#### Identification of haplotypes that carry putative recessive lethal or semi-lethal alleles

Haplotypes that show absence or a reduced level of homozygosity at *p *< 5 × 10^−8^ (−log_10_*p *> 7.30) were considered as candidates for carrying putatively recessive lethal or semi-lethal alleles that cause early embryonic death or reduced postnatal survival. In order to decrease the false discovery rate, only 1 Mb long regions with at least nine haplotype tests with *p *< 5 × 10^−8^ were considered. From each of these regions, only the most significant haplotype test from each of the three test categories was selected. We analysed the effect of each of these haplotypes on cow reproduction and progeny survival to corroborate lethality and on production traits to evaluate pleiotropy.

### Reproduction and survival analysis to corroborate the identified haplotypes

We used routine cow reproduction and progeny survival records from the ICBF database. We tracked 44,351 insemination records that pertain to purebred Aberdeen Angus, Charolais, Hereford, Limousin, or Simmental cattle, for which both the cow and the AI sire that it is mated to, were genotyped. There were 7540 records for Aberdeen Angus, 11,520 for Charolais, 5566 for Hereford, 15,404 for Limousin, and 4321 for Simmental. Altogether these inseminations resulted in 21,196 progeny. Each resulting calf had records for its date of birth and, if deceased, its date of death and the cause of death (natural death or slaughtered). All the new born calves were followed from birth until November 2017. If they were still alive at the end of data collection their records were treated as censored.

To quantify the effect of identified haplotypes on reproduction, we determined the insemination success rate based on the number of days between a recorded insemination and the subsequent calving date. Insemination success rate was calculated as a ratio between the number of born progeny and the number of inseminations. These statistics were reported for the three mating types: (1) *Hh *× *Hh*, (2) *Hh *× *HH*, and (3) *HH *× *HH*. The interval between insemination and calving was calculated from 40,769 records for which the date of the corresponding calving was known. In order to accommodate shorter or longer than average gestation length, a window of 7, 14, or 21 days was allowed on either side of the breed average gestation length, calculated as the average gestation length for the set of individuals recorded for that breed. If a calving occurred outside of the window, the insemination was deemed to be unsuccessful. If a haplotype harbours recessive lethal alleles, *Hh *× *Hh* matings are expected to have a 25% lower insemination success since there will be 25% *hh* progeny and an interval between insemination and calving longer than 21 days, which is the average time between each standing oestrus in cattle.

To quantify the effect of identified haplotypes on postnatal survival, we used survival analysis with the Cox proportional-hazard model. We compared hazard ratios between the progeny from: (1) *Hh *× *Hh*, (2) *Hh *× *HH*, and (3) *HH *× *HH* matings. If a haplotype affects postnatal survival, we would expect *hh* progeny from *Hh *× *Hh* matings to have a higher hazard ratio. Since two causes of death were reported (natural death and slaughtered), we analysed data in two ways: (1) we treated records from slaughtered animals as censored, and (2) we treated their records as uncensored.

### Pleiotropy analysis

We analysed estimated breeding values to investigate pleiotropic effects of the identified haplotypes on economically important traits in beef production. Estimated breeding values for 15 traits (feed intake, live weight, cull cow weight, carcass weight, carcass confirmation, carcass fat, docility, gestation length, age at first calving, calving interval, calving difficulty due to the sire, maternal calving difficulty, mortality at calving, maternal weaning weight, and cow survival) for all genotyped individuals in this study were available from the May 2017 routine genetic evaluation carried out by the ICBF. These 15 traits are included in two indexes with different economic weights and trait emphasis (shown in parentheses). The economic weights were calculated based on the costs of inputs and the value of final products [24]. We used the same economic weights and trait emphasis as used currently by ICBF. The terminal index consists of: calving difficulty due to sire (− €4.65, 18%), gestation length (− €2.25, 4%), mortality (− €5.34, 3%), docility (€17.03, 2%), feed intake (-€0.10, 16%), carcass weight (€3.14, 41%), carcass conformation (€14.77, 11%), and carcass fat (− €7.86, 5%). The replacement index consists of eight cow traits: maternal calving difficulty (− €4.98, 6%), age at first calving (− €0.99, 6%), calving interval (− €5.07, 9%), cow survival (€8.86, 8%), maternal weaning weight (€5.58, 18%), cow live weight (− €1.31, 13%), cow docility (€77.27, 4%), cull cow weight (€0.91, 7%); and eight calf traits: calving difficulty due to sire (− €5.12, 7%), gestation length (− €2.48, 2%), mortality (− €5.87, 1%), calf docility (€14.72, 1%), feed intake (− €0.07, 4%), carcass weight (€2.10, 10%), carcass conformation (€10.22, 3%), and carcass fat (− €5.44, 1%). For every identified haplotype, we estimated its substitution effect on the estimated breeding values for each of the 15 traits and two indexes. Only estimated breeding values with at least 25% reliability on the trait of interest were used, which amounted to between 1515 and 43,963 records being used of which between 29.4 and 87.0% were from sires with progeny and between 0.8 and 92.4% were from animals with their own phenotypes. The substitution effect was estimated by linear regression of estimated breeding values on the number of haplotype copies that an animal carries. Trait-specific substitution effects were divided by the standard deviation of the estimated breeding values to present the effects on a comparable scale.

### Economic impact

For the estimation of economic impact of putative lethal or semi-lethal haplotypes on the whole Irish beef population, we built a simple economic model. This model was based on the number of purebred sire × purebred dam, purebred sire × crossbred dam, and crossbred sire × crossbred dam of Aberdeen Angus, Charolais, and Simmental animals, the expected number of progeny genotypes, haplotype frequencies, insemination success rate, number of days between the subsequent inseminations, daily costs for maintaining a cow, and substitution effects. We obtained these inputs from this study, the ICBF database, and the E-profit monitor—an online financial analysis tool [25].

### Candidate genes

We used Bioconductor version 3.7 with the BioMart database version Ensembl Genes 92 using the btaurus_gene_ensembl to find candidate genes within the regions of the identified haplotypes [26]. We used the UMD3.1 cattle reference genome [27]. We extracted the protein coding genes and searched for lethal effects observed with homologous genes with the same function in the Mouse Genome Informatics database [28].

## Results

Our results identified one haplotype that carries a putatively recessive lethal allele and two haplotypes that carry putatively recessive semi-lethal alleles, which were located on chromosome 16 in Simmental, chromosome 14 in Aberdeen Angus, and chromosome 19 in Charolais, respectively. These haplotypes showed pleiotropic effects on economically important beef production traits. We identified the *zinc finger and AT*-*hook domain containing* (*ZFAT*) gene as the potential causative gene for semi-lethality in Aberdeen Angus.

### Identification of haplotypes that carry putative recessive lethal or semi-lethal alleles

#### Haplotype tests

In total, there were between 36 and 109 haplotypes with significant absence or reduced levels of homozygosity at *p *< 5 × 10^−8^ in the five breeds. Table 2 shows the number of significant haplotypes by test and breed. The largest number of significant haplotypes was observed for Limousin (109) followed by Charolais (86), Aberdeen Angus (69), Hereford (57), and Simmental (36). Among the different tests, the semi-lethality test on sire × dam matings had the largest number of significant haplotypes. The carrier mating test on sire × dam matings had the second largest number of significant haplotypes for Charolais, Hereford, and Simmental.

**Table 2:** Number of haplotypes with a significant absence or reduced level of homozygosity at *p *< 5 × 10^−8^ by test and breed

Test^a^	Aberdeen Angus	Charolais	Hereford	Limousin	Simmental
T1	3	0	0	8	6
T2	1	9	13	14	15
T3	2	3	0	3	1
T4	65	72	56	90	16
T5	1	5	11	15	1
Total^b^	69	86	57	109	36

^a^T1: population test; T2: carrier mating test on sire × dam matings; T3: carrier mating test on sire × maternal grand-sire matings; T4: semi-lethality test on sire × dam matings; T5: semi-lethality test on sire × maternal grand-sire matings

^b^Total number of haplotypes with *p *< 5 × 10^−8^ from at least one test

#### Identification of haplotypes that carry putative recessive lethal or semi-lethal alleles

**Fig. 1:**
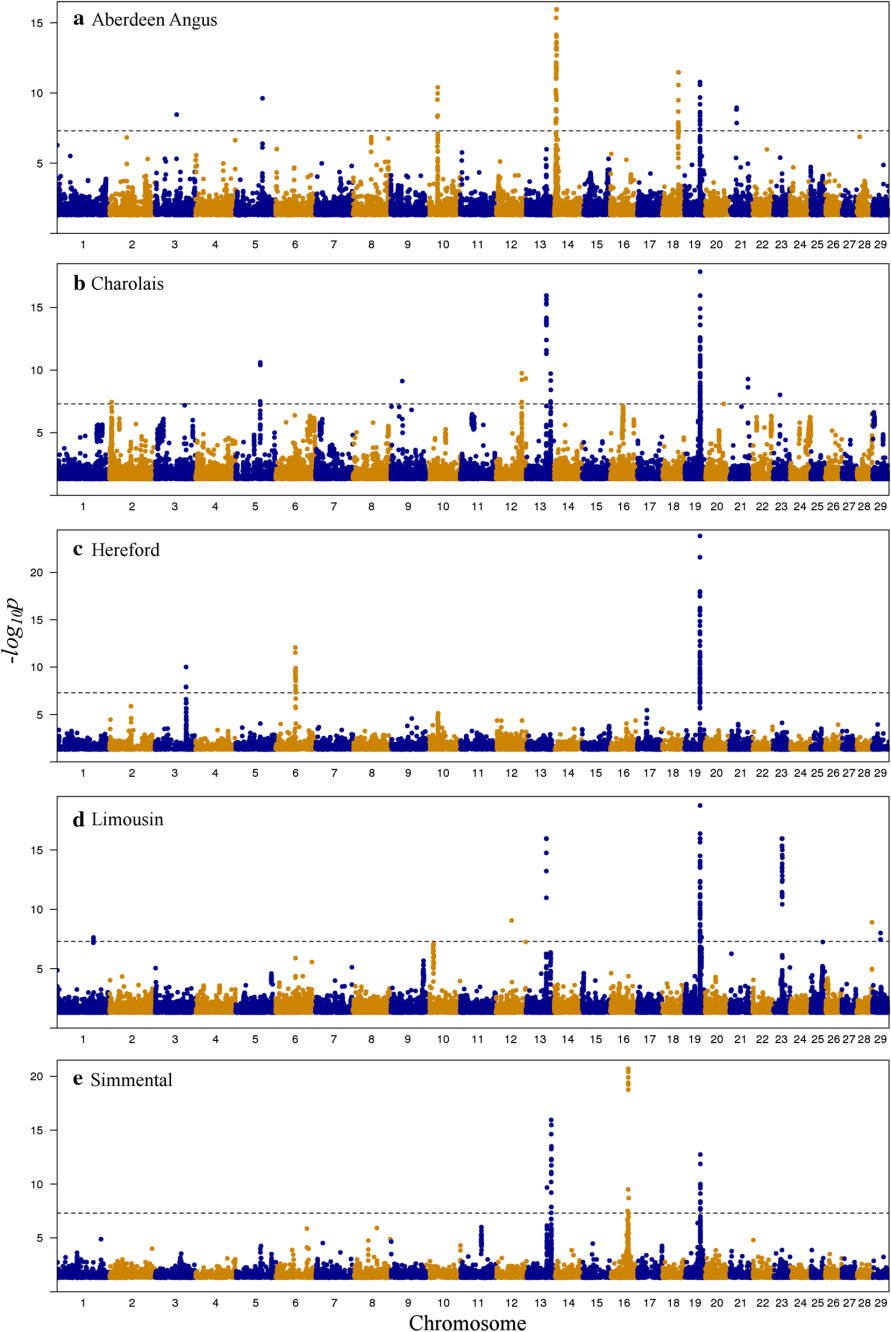
Genome-wide Manhattan plot for lack of homozygosity in **a** Aberdeen Angus, **b** Charolais, **c** Hereford, **d** Limousin, and **e** Simmental breeds

Across all five breeds, there were 19 one-Mb long regions with at least nine haplotypes that showed significant absence or a reduced level of homozygosity. These were considered as candidate regions with haplotypes that carry putatively recessive lethal or semi-lethal alleles. Figure 1 shows the negative logarithm of all test p-values across the whole genome and the 19 candidate regions are listed in Additional file 1: Table S1, with five regions for Aberdeen Angus, two for Charolais, three for Hereford and Limousin, and six for Simmental. Some of the regions were in close proximity and appeared as a single region (see Fig. 1). This was the case for three regions on chromosome 14 in Aberdeen Angus, two regions on chromosome 6 in Hereford, two regions on chromosome 23 in Limousin, and three regions on chromosome 13 and two regions on chromosome 16 in Simmental. The region between 48 and 49 Mb on chromosome 19 was detected in all the breeds.

From the 19 candidate regions, we selected 22 haplotypes with the highest significance within each of the three category tests. Table 3 lists the 22 haplotypes and their characteristics. We named the haplotypes by using a code including the breed (AA for Aberdeen Angus, CH for Charolais, HE for Hereford, LI for Limousin and SI for Simmental), the chromosome, and the haplotype number within the breed (H for haplotype). Except for HE19H3 and SI19H6, which were identified with tests from two categories (carrier mating test with sire × dam matings from the carrier mating tests and semi-lethality test with sire × dam matings from the semi-lethality tests), all haplotypes were identified by tests from a single category.

**Table 3:** Identified haplotypes carrying putatively recessive lethal or semi-lethal alleles

Haplotype name	Test^a^	Chr^b^	Haplotype region	Sliding window	Start base	End base	Haplotype percentage
AA14H1	T4	14	AA14R1	4	6,819,966	7,514,610	25.2
AA14H2	T4	14	AA14R2	9	7,646,485	8,425,401	15.3
AA14H3	T4	14	AA14R3	20	8,064,004	8,927,881	15.2
AA14H4	T3	14	AA14R3	9	8,454,690	9,215,622	15.3
AA18H5	T4	18	AA14R4	4	49,446,631	50,616,688	18.9
AA19H6	T4	19	AA14R5	8	48,850,043	48,874,999	23.9
CH13H1	T4	13	CH13R1	19	61,565,261	61,584,644	33.5
CH19H2	T4	19	CH19R2	5	48,874,999	48,902,319	14.4
CH19H3	T3	19	CH19R2	12	48,889,025	48,914,435	1.2
HE6H1	T4	6	HE6R1	8	58,473,731	59,706,074	30.2
HE6H2	T4	6	HE6R2	4	59,688,857	60,919,136	28.8
HE19H3	T2, T4	19	HE19R3	13	48,854,119	48,900,428	28.9
LI19H1	T5	19	LI19R1	20	48,851,566	48,871,260	12.7
LI19H2	T2	19	LI19R1	11	48,883,348	48,906,682	11.1
LI23H3	T4	23	LI23R2	20	27,923,154	28,649,349	13.7
LI23H4	T4	23	LI23R3	12	28,307,706	29,872,481	11.5
SI13H1	T2	13	SI13R1, SI13R2	15	73,746,516	74,973,171	9.5
SI13H2	T4	13	SI13R1, SI13R2, SI13R3	19	73,895,443	75,240,419	22.3
SI13H3	T2	13	SI13R2, SI13R3	1	74,109,992	75,310,875	9.3
SI16H4	T2	16	SI16R4, SI16R5	16	51,195,450	53,097,936	10.8
SI16H5	T1	16	SI16R4, SI16R5	4	51,811,400	53,434,159	8.8
SI19H6	T2, T4	19	SI19R6	18	48,873,060	48,891,361	18.4

^a^T1: population test; T2: carrier mating test on sire × dam matings; T3: carrier mating test on sire × maternal grand-sire matings; T4: semi-lethality test on sire × dam matings; T5: semi-lethality test on sire × maternal grand-sire matings

^b^Chr: chromosome number

### Reproduction and survival analysis to corroborate the identified haplotypes

#### Reproduction analysis

Two out of the 22 identified haplotypes, AA14H3 and SI16H5, were associated with decreased insemination success rate and a longer interval between insemination and calving. Table 4 shows the insemination success rate and the length of the interval between insemination and calving for the non-carrier × non-carrier (*HH *× *HH)*, non-carrier × carrier (*HH *× *Hh)*, and carrier × carrier (*Hh *× *Hh)* matings for the AA14H3 and SI16H5 haplotypes. AA14H3 was located on chromosome 14 (8,064,004–8,927,881 bp) in Aberdeen Angus and SI16A5 on chromosome 16 (51,811,400–53,434,159 bp) in Simmental. The insemination success rates for *Hh *× *Hh* matings were 24.2, 23.0, and 20.2% lower (AA14H3), and 48.6, 52.5, and 52.7% lower (SI16H5) compared to the *HH *× *HH* matings for windows of 7, 14, and 21 days on either side of the average gestation length for each breed, respectively. The insemination success rates were also lower in *HH *× *Hh* matings than in *HH *× *HH* matings, but the differences were smaller.

**Table 4:** Insemination success rate and interval between insemination and calving for non-carrier × non-carrier matings (*HH *× *HH*), non-carrier × carrier (*HH *× *Hh*), and carrier × carrier (*Hh *× *Hh*) matings for the AA14H3 and SI16H5 haplotypes and a calf registration window of 7, 14, and 21 days

Mating type	Window (days)	Haplotype allele					
		AA14H3 (8,064,004–8,927,881 bp)^a^			SI16H5 (51,811,400–53,434,159 bp)^a^		
		*HH* × *HH*	*HH* × *Hh*	*Hh *×* Hh*	*HH* × *HH*	*HH* × *Hh*	*Hh *×* Hh*
Number of matings		1005	783	154	779	492	32
Success rate (%)	7	42.9	41.3	32.5	36.6	35.0	18.8
	14	54.0	51.6	41.6	46.1	44.5	21.9
	21	55.4	53.9	44.2	46.3	44.7	21.9
Change in success rate against non-carrier × non-carrier matings (%)	7		− 3.7	− 24.2		− 4.4	− 48.6
	14		− 4.4	− 23.0		− 3.5	− 52.5
	21		− 2.7	− 20.2		− 3.5	− 52.7
Days from insemination to calving		319	323	349	328	337	358

^a^Genomic region in bp of the haplotype

For both haplotypes (AA14H3 and SI16H5) the interval between insemination and calving was longer for *Hh *× *Hh* matings than for *HH *× *Hh* matings, which was in turn longer than for *HH *× *HH* matings. The interval between insemination and calving for *Hh *× *Hh* matings was 30 days longer than for *HH *× *HH* matings for both AA14H3 and SI16H5 haplotypes, and that for *HH *× *Hh* matings was longer than for *HH *× *HH* matings by 4 days for AA14H3 and by 9 days for SI16H5.

#### Survival analysis

One of the identified haplotypes, CH19H2 was associated with a significantly (p < 0.05) increased hazard ratio for postnatal survival (i.e. decreased survival) of progeny from *Hh *× *Hh* matings. CH19H2 is located on chromosome 19 (48,874,999–48,902,319 bp) in Charolais. Additional file 2: Table S2 presents the hazard ratios for progeny from *HH *× *Hh* and *Hh *× *Hh* matings compared to those for progeny from *HH *× *HH* matings, for which records for culled progeny were treated as censored or complete. Progeny of individuals that carried the CH19H2 haplotype had a 36% higher probability of dying or being slaughtered during their life compared to the progeny of *HH *× *HH* parents.

#### Frequencies for haplotypes that carry putatively recessive lethal or semi-lethal alleles

All three haplotypes that carry putatively recessive lethal or semi-lethal alleles associated with decreased insemination success or reduced postnatal survival had high frequencies. Table 5 summarizes the statistics for the AA14A3, CH19A2, and SI16A5 haplotypes. Haplotype frequencies were 15.2% for AA14A3, 14.4% for CH19A2, and 8.8% for SI16A5. Ninety-five recessive homozygous individuals were observed for AA14A3, 83 for CH19A2, and none for SI16A5. This suggests that the SI16A5 haplotype carries a putatively recessive lethal allele, whereas AA14A3 and CH19A2 haplotypes carry putatively recessive semi-lethal alleles. Table 5 shows that the number of observed recessive homozygotes was always smaller than the expected number for different types of comparisons. SNP alleles present on the AA14A3, CH19A2, and SI16A5 haplotypes are in Additional file 3: Table S3.

**Table 5:** Statistics for the AA14H3, CH19H2, and SI16H5 haplotypes

Haplotype	AA14H3	CH19H2	SI16H5
Genotyped animals	22,510	38,960	11,559
Frequency (%)	15.2	14.4	8.8
Recessive homozygotes	95	83	0
Expected recessive homozygotes	194.1	256	47.6
Sire-carrier × dam-carrier matings	220	256	17
Recessive homozygotes from sire-carrier × dam-carrier matings	2	6	0
Expected recessive homozygotes from sire-carrier × dam-carrier matings	55	64	4.3
Sire-carrier × maternal grand-sire-carrier matings	141	87	7
Recessive homozygotes from sire-carrier × maternal grand-sire-carrier matings	0	1	0
Expected recessive homozygotes from sire-carrier × maternal grand-sire-carrier matings	17.6	10.9	0.9
Probability of not observing recessive homozygotes with the T1 test^a^	2.3 × 10^−85^	2.5 × 10^−112^	2.0 × 10^−21^
Probability of not observing recessive homozygotes with the T2 test^a^	3.3 × 10^−28^	1.0 × 10^−32^	7.9 × 10^−3^
Probability of not observing recessive homozygotes with the T3 test^a^	6.7 × 10^−9^	9.0 × 10^−9^	3.9 × 10^−1^
Probability that the observed and expected number of recessive homozygotes are not different with the T4 test^a^	1.1 × 10^−16^	1.1 × 10^−16^	1.7 × 10^−2^
Probability that the observed and expected number of recessive homozygotes are not different with the T5 test^a^	7.2 × 10^−6^	1.4 × 10^−3^	3.2 × 10^−1^

^a^T1: population test; T2: carrier mating test on sire × dam matings; T3: carrier mating test on sire × maternal grand-sire matings; T4: semi-lethality test on sire × dam matings; T5: semi-lethality test on sire × maternal grand-sire mating

### Pleiotropy analysis

**Fig. 2:**
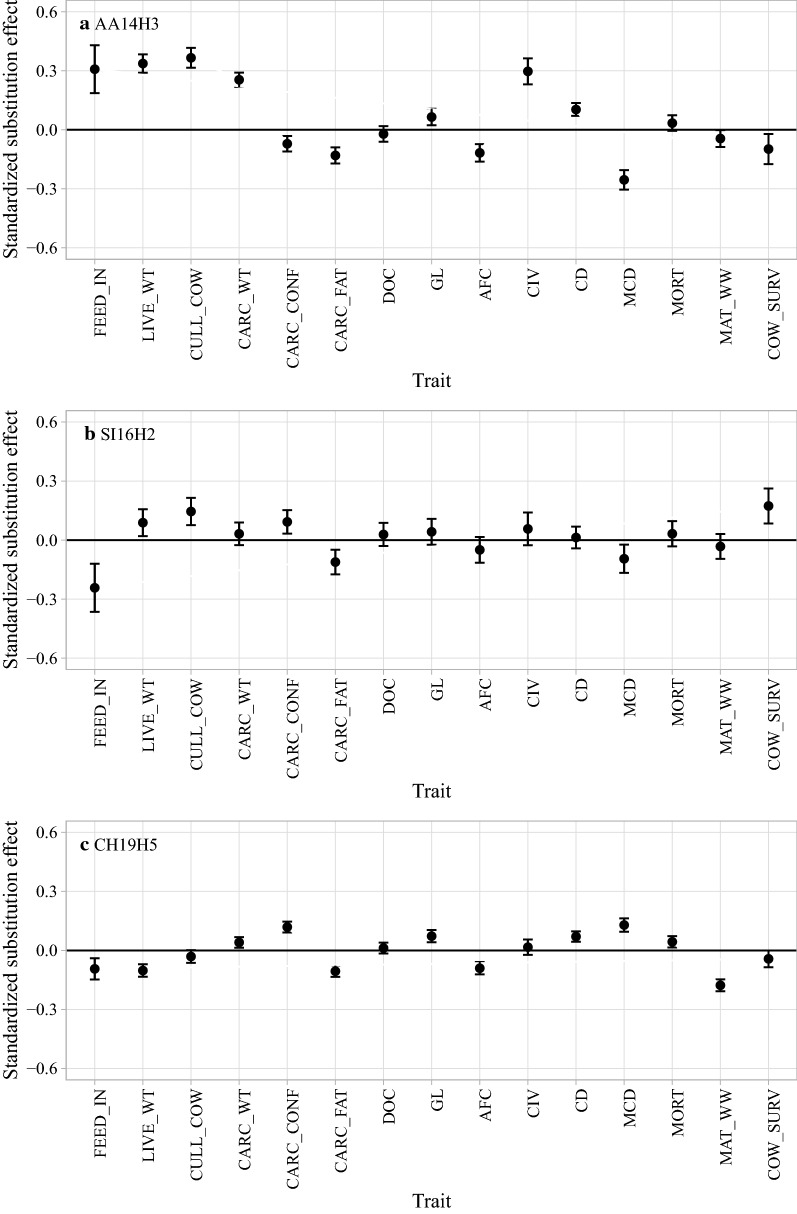
Standardized substitution effects with 95% confidence interval for the haplotypes **a** AA14H3, **b** SI16H5, and **c** CH19H2 on 15 traits. FEED_IN, feed intake; LIVE_WT, live weight; CULL_COW, cull cow weight; CARC_WT, carcass weight; CARC_CONF, carcass confirmation; CARC_FAT, carcass fat; DOC, docility; GL, gestation length; AFC, age at first calving; CIV, calving interval; CD, calving difficulty due to the sire; MCD, maternal calving difficulty; MORT, mortality at calving; MAT_WW, maternal weaning weight; COW_SURV, cow survival

To investigate potential pleiotropic effects on traits under selection, we estimated the substitution effects of the AA14H3, CH19H2, and SI16H5 haplotypes. Figure 2 shows standardized substitution effects, presented in the units of a trait standard deviation, for 15 traits that are part of the terminal or replacement index. A significant substitution effect for the AA14H3 haplotype was observed for cull cow weight (0.37), followed by live weight (0.34), feed intake (0.31), calving interval (0.30), maternal calving difficulty (− 0.25), carcass weight (0.25), carcass fat (− 0.13), age at first calving (− 0.12), calving difficulty (0.10), cow survival (− 0.10), carcass conformation (− 0.07), gestation length (0.06), and maternal weaning weight (− 0.04). Collectively, one copy of the AA14H3 haplotype increased the terminal index by €3.42 where one standard deviation is €18.65, and decreases the replacement index by €3.11, where one standard deviation is €29.82. A significant substitution effect for the SI16H5 haplotype was observed for feed intake (− 0.24), followed by cow survival (0.17), cull cow weight (0.15), carcass fat (− 0.11), maternal calving difficulty (− 0.09), carcass conformation (0.09), and live weight (0.09). Collectively, one copy of the SI16H5 haplotype increased the terminal index by €2.30 where one standard deviation is €22.52 and the replacement index by €1.03 where one standard deviation is €31.35. A significant substitution effect for the CH19H2 haplotype was observed for maternal weaning weight (− 0.18), maternal calving difficulty (0.13), carcass conformation (0.12), carcass fat (− 0.11), live weight (− 0.10), feed intake (− 0.09), age at first calving (− 0.09), gestation length (0.07), calving difficulty (0.07), mortality (0.04), and carcass weight (0.04). Collectively, one copy of the CH19H2 haplotype increased the terminal index by €1.47 where one standard deviation is €22.70 and decreased the replacement index by €0.88 where one standard deviation is €35.79.

### Economic impact

The expected economic impact estimated with a simple economic model is presented in Additional file 4: Table S4. The annual losses due to decreased viability and decreased survival for each haplotype were estimated to reach ~ €570,000 for AA14H3, ~ €930,000 for CH19H2, and ~ €210,000 for SI16H5. The annual gains due to positive associations with economically important traits were estimated to reach ~ €110,000 for AA14H3, ~ €70,000 for CH19H2, and ~ €50,000 for SI16H5. Under the current population structure in Ireland, the estimated national annual net effects were negative: − €450,000 for AA14H3 − €860,000 for CH19H2, and − €160,000 for SI16H5. The large negative net effect of the CH19H2 haplotype is due to the larger purebred population in Charolais than in Aberdeen Angus and Simmental. The economic model shows that, under the current population structure, the maximum annual net effects would be achieved with haplotype frequencies of 0.02% for AA14H3 (+€7,000), 0.01% for CH19H2 (+€2,000), and SI16H5 (+€4,000) [see Additional file 5: Figure S1].

### Candidate genes

We identified one protein coding gene in the region of AA14H3, none in the region of CH19H2, and several in the region of SI16H5. *Zinc finger and AT*-*hook domain containing* (*ZFAT*) is the only protein coding gene located between 8,064,004 and 8,927,881 bp on chromosome 14 of the AA14A3 haplotype. *ZFAT* is associated with prenatal or perinatal lethality in the Mouse Informatics Database. Additional file 6: Table S5 lists the 64 protein coding genes located between 51,811,400 and 53,434,159 bp on chromosome 16 of the SI16H5 haplotype, among which five, i.e. *SKI proto*-*oncogene* (*SKI*), *G protein subunit beta 1* (*GNB1*), *ATPase family AAA domain*-*containing protein 3* (*ATAD3A*), *zinc finger and BTB domain containing 17* (*ZBTB17*), and *caspase*-*9* (*CASP9*)) are associated with prenatal or perinatal lethality in mice.

## Discussion

Our results reveal one haplotype that carries a putatively recessive lethal allele (SI16A5) and two haplotypes that carry putatively recessive semi-lethal alleles (AA14A3 and CH19A2) that are candidates for lethality or semi-lethality in homozygous state. These three haplotypes were identified by the haplotype analysis of 132,725 purebred animals of five beef cattle breeds from the ICBF database. The results also suggest pleiotropy, since these haplotypes include or are in strong linkage with variants that have substantial effects on economically important traits. In the light of these results, we discuss (1) the identification of haplotypes that carry putative recessive lethal and semi-lethal alleles, (2) the frequency of the identified haplotypes, (3) their economic impact, and (4) their potential genetic causes.

### Identification of haplotypes that carry putative recessive lethal or semi-lethal alleles

We used five tests on sliding haplotypes and phenotypic information to search for haplotypes that carry recessive lethal or semi-lethal alleles. We used haplotypes in a sliding window of 20 SNPs, which was previously shown to be a good compromise between haplotype diversity and detection of putative recessive lethal alleles with a moderate SNP density array [15]. The tests were based on the number of expected recessive homozygous individuals either in the whole population or from matings between carriers. Along with the tests for complete absence of recessive homozygotes, we also used a set of tests that allowed for some recessive homozygotes but tested whether their number was significantly smaller than expected. This allowed us to take possible incomplete penetrance, incomplete linkage between haplotype and putative recessive lethal alleles, structural variation, or genotyping, phasing, and imputation errors into account. In the following, we consider that all are semi-lethal.

The top 22 candidates from the haplotype analysis were further corroborated with cow insemination and progeny survival analysis, and three haplotypes showed phenotypic evidence of lethality. Recessive lethal alleles are expected to decrease insemination success and extend the interval between insemination and calving. In the case of semi-lethality, some embryos are expected to survive, but these may have a decreased viability that will in turn affect their survival.

Although there were many haplotypes with a significant absence or reduced level of homozygosity, which indicated that they carried putatively recessive lethal or semi-lethal alleles, only two haplotypes were supported by the reproduction analysis. In particular, there was a strong signal observed at the end of chromosome 19 for all five cattle breeds, which was supported by phenotypic data only in the Charolais breed. A highly significant departure from Hardy–Weinberg equilibrium was observed for SNPs that were located at the end of chromosome 19 with an excessive heterozygosity level (results not shown). This may indicate genotyping errors or structural variation in this region [29] that could explain the strong signal observed.

### Frequency of haplotypes that carry putatively recessive lethal or semi-lethal alleles

For our methods to detect a haplotype that carries putatively recessive lethal or semi-lethal alleles, we needed a relatively high haplotype frequency or many *Hh *× *Hh* matings. Because most haplotypes are rare, this means that the methods we used had sufficient power for a very small proportion of the haplotypes. Following the probability calculations of the different tests and fixing the number of genotyped animals to the number available for this study, we were able to assess the minimum required haplotype frequency with a significance of *p *< 5 × 10^−8^, i.e. 5.42% for Aberdeen Angus, 4.14% for Charolais, 7.29% for Hereford, 3.83% for Limousin, and 7.56% for Simmental. The percentages of haplotypes with a frequency higher than these thresholds were very low: 0.98% for Aberdeen Angus, 0.68% for Charolais, 1.01% for Hereford, 0.56% for Limousin, and 0.52% for Simmental. Similarly, reaching such significance with the carrier mating test would require at least 59 sire × dam carrier (*Hh *× *Hh*) matings and no homozygous progeny. The percentages of haplotypes reaching this threshold were even smaller than for the population test: 0.44% for Aberdeen Angus, 0.23% for Charolais, 0.37% for Hereford, 0.20% for Limousin, and 0.16% for Simmental. Furthermore, reaching significance with the carrier mating test would require 126 sire × maternal grand-sire carrier (*Hh *× *Hh)* matings and no homozygous progeny. The percentage of haplotypes reaching this threshold were even smaller than for the carrier mating test with sire × dam matings, i.e. 0.06% for Aberdeen Angus, 0.04% for Charolais and Hereford, 0.03% for Limousin, and 0.02% for Simmental. Finally, reaching significance with the semi-lethality test required that the Chi square test statistic be higher than 29.72. The percentages of haplotypes that reached this threshold in the current study was less than 0.01% for all the breeds.

Since recessive lethal and semi-lethal alleles are usually rare and breed-specific, they are difficult to detect in a population, in particular for recent mutations since modern breeding programs try to avoid inbreeding to preserve genetic variation and maintain fitness. Thus, breeders are likely to indirectly avoid carrier matings because they avoid matings between close relatives. Still, there are some alleles that are propagated through the population via popular sires, have pleiotropic effects, or are in incomplete linkage with an allele that has a positive effect on a trait that was selected for in the past. A classic example of a recessive lethal allele propagated with a popular sire is the complex vertebral malformation (CVM) of calves in the Holstein breed. The causal mutation was propagated by the bull Carlin-M Ivanhoe Bell that was incidentally also a carrier of the bovine leukocyte adhesion deficiency (BLAD) [30]. An example of a pleiotropic recessive lethal allele is a 660-kb deletion that causes embryonic death in Nordic Red cattle [14]. Since this deletion is positively associated with milk yield, it is quite common (13–32%) in the Nordic Red cattle population. Yet another example is the Weaver syndrome which is expressed postnatally between the age of 5 and 8 months [31], with Weaver-carriers also producing more milk and fat, which have been strongly selected for in the past [31, 32].

High haplotype frequencies and substitution effects on traits under selection suggest that the frequency of the detected haplotypes in this study may have increased because of pleiotropy or linked selection. The observed frequencies are high compared to expected frequencies with mutation-selection equilibrium. We can use Nei’s [33] formula to approximate the expectation and variance of the frequency of a recessive lethal allele in a finite population, where *µ* is the mutation rate, *N*_*e*_ the effective population size, and *s* the selection coefficient against the mutant lethal:

$$\bar{q} \approx \mu \sqrt {2 \pi \frac{{N_{e} }}{s}} \quad {\text{and}}\quad \sigma^{2} \approx \frac{\mu }{s} - \bar{q}^{2} .$$

With a high mutation rate of *µ *= 10^−5^ and an *N*_*e*_ similar to that of some beef cattle populations of 300, e.g. [34, 35], the expected equilibrium frequency is very low, regardless of whether the lethal allele is fully (*s* = 1) or partially penetrant (*s* = 0.75): $\bar{q} \approx 0.0004$ and *σ *≈ 0.0031 for a fully penetrant lethal allele, and $\bar{q} \approx 0.0005$ and *σ *≈ 0.0036 for a partially penetrant lethal allele. This analysis clearly shows that there are other forces, i.e., selection, that maintain the high frequency of the three haplotypes.

### Economic impact

Analysis of the economic impact of the three haplotypes on the whole Irish beef population supports the role of pleiotropy in maintaining high haplotype frequencies, which should be managed with appropriately optimised breeding programs. The economic impact could be increased if haplotype carriers are identified and mating between carriers is avoided. This is relatively easy to achieve when there is only one or a few haplotypes for which carrier matings are to be avoided. However, if there are many lethal haplotypes to select against, it will be much more difficult to achieve within pure breeds. Crossbreeding could overcome this issue for breed-specific haplotypes.

### Genetic causes of lethality and pleiotropy

We found one candidate gene for one haplotype that carries a putatively recessive lethal allele, no candidate genes for another, and several for the third one. *ZFAT* was the only protein coding gene that overlapped with the AA14H3 haplotype. ZFAT is a transcription factor involved in immune-regulation and apoptosis [36, 37] and plays a role in the development of the hematopoietic system [38]. The Mouse Genome Informatics database reported complete early embryonic lethality for a knock-out allele of *ZFAT*. *ZFAT* is also known to be associated with human height [39–41] and body size in horses [42]. This is consistent with our results, since we observed an insemination success rate that was 20.2 to 24.2% lower (based on different gestation window size) for the AA14H3 *Hh *× *Hh* matings than for the HH × HH matings, and the haplotype was positively associated with weight-related traits and feed intake.

Sixty-four protein coding genes overlapped with the SI16H5 haplotype, of which five *SKI*, *GNB1*, *ATAD3A ZBTB17*, and *CASP9* were associated with prenatal or perinatal lethality in the Mouse Genome Informatics database [43–47]. This haplotype displays a positive association with feed conversion ratio, which may explain its high frequency in the population.

There were no homozygous individuals for the SI16H5 haplotype, which suggests that the identified haplotype tags the putative recessive lethal allele(s). The existence of homozygous individuals for the AA14H3 and CH19H2 haplotypes suggests that there may be multiple underlying haplotypes with and without lethal allele(s), but we are unable to distinguish them with the current genotyping data. Another possible reason is incomplete penetrance, resulting in some homozygotes not suffering from the deleterious effects of the allele.

Fine-mapping these haplotypes with sequence data could potentially resolve the fine haplotype structure, and identify causal variants for lethality and pleiotropic effects on the selected traits. Efforts to identify such causative variants are ongoing.

## Conclusions

We identified three haplotypes, one that carries a putatively recessive lethal allele in Aberdeen Angus and two that carry semi-lethal alleles, one in Charolais and one in Simmental. Carrier matings in Aberdeen Angus and Simmental had a lower success of artificial insemination and a longer interval between insemination and calving. Progeny from carrier matings in Charolais had a higher probability of dying or culling. The haplotype in Aberdeen Angus was in a region that contains the *ZFAT* gene, which was previously shown to be lethal in mice. The haplotype in Simmental was in a region that contains several candidate genes that potentially cause prenatal or perinatal lethality. Substitution analysis indicated that the haplotypes had pleiotropic effects on the traits under selection. The substitution effects were positive for the terminal index and positive to negative for the replacement index. Further work is needed to identify the underlying causative variants that impact the traits under selection and fitness. Identification of haplotype carriers will improve the breeding success and fitness of beef cattle populations.

## Additional files

**Additional file 1: Table S1.** Genomic regions with at least nine significant haplotypes for absence or reduced level of homozygosity at *p *< 5 × 10^−8^ by breed. The data provided describe genomic location of genomic regions with at least nine significant haplotypes for the absence or reduced level of homozygosity.

**Additional file 2: Table S2.** Hazard ratios [95% confidence intervals] for postnatal survival of progeny from non-carrier × carrier (*HH *× *Hh*) and carrier × carrier (*Hh *× *Hh*) matings as compared to the progeny from non-carrier × non-carrier (*HH *× *HH*) matings for the 22 identified haplotypes with significant absence or reduced level of homozygosity. The data provided represent survival analysis for different parent haplotype carrier status for several haplotypes with significant absence or reduced level of homozygosity.

**Additional file 3: Table S3.** SNP alleles for the AA14H3, CH19H2, and SI16H5 haplotypes. The data provided represent the SNP alleles for haplotype (SI16H5) that carries putatively recessive lethal and two haplotypes (AA14H3, CH19H2) that carry semi-lethal alleles.

**Additional file 4: Table S4.** Estimated economic effect for the AA14H3, CH19H2, and SI16H5 haplotypes. The data provided represent estimated economic impact for haplotype (SI16H5) that carries putatively recessive lethal and two haplotypes (AA14H3, CH19H2) that carry semi-lethal alleles.

**Additional file 5: Figure S1.** Estimated national annual net effect for the AA14H3, CH19H2, and SI16H5 haplotypes with vertical lines showing the net effect under the current haplotype frequencies. The figure provided shows the net effect for haplotype (SI16H5) that carries putatively recessive lethal and two haplotypes (AA14H3, CH19H2) that carry semi-lethal alleles for different haplotype frequencies.

**Additional file 6: Table S5.** Protein coding genes between 51,611,400 and 53,234,159 bp on bovine chromosome 16 for the SI16H5 haplotype; the genes showing prenatal or perinatal lethality in mice are in bold. The data provided represent protein coding genes located on the haplotype (SI16H5) that carries putatively recessive lethal allele.
