# Supplementary material for: Analysis of a large dataset reveals haplotypes carrying putatively recessive lethal and semi-lethal alleles with pleiotropic effects on economically important traits in beef cattle
Source: Genet Sel Evol. 2019 Mar 5;51:9. doi: 10.1186/s12711-019-0452-z (PMC6402105; doi:10.1186/s12711-019-0452-z)
Supplement: Supplementary file 1 — Additional file 1: Table S1. Genomic regions with at least nine significant haplotypes for absence or reduced level of homozygosity at p < 5 × 10^−8^ by breed. The data provided describe genomic location of genomic regions with at least nine significant haplotypes for the absence or reduced level of homozygosity. [file 12711_2019_452_MOESM1_ESM.docx]

| **Breed** | **Region name** | **Chromosome** | **Start (Mb)** | **End (Mb)** | **Number of haplotypes** | **Test category^1^** |
| --- | --- | --- | --- | --- | --- | --- |
| Aberdeen Angus | AA14R1 | 14 | 6 | 7 | 10 | 3 |
|  | AA14R2 | 14 | 7 | 8 | 24 | 3 |
|  | AA14R3 | 14 | 8 | 9 | 22 | 2, 3 |
|  | AA18R4 | 18 | 49 | 50 | 9 | 3 |
|  | AA19R5 | 19 | 48 | 49 | 12 | 3 |
| Charolais | CH13R1 | 13 | 61 | 70 | 14 | 3 |
|  | CH19R2 | 19 | 48 | 50 | 49 | 2, 3 |
| Hereford | HE6R1 | 6 | 59 | 60 | 16 | 3 |
|  | HE6R2 | 6 | 60 | 61 | 9 | 3 |
|  | HE19R3 | 19 | 48 | 49 | 42 | 2, 3 |
| Limousin | LI19R1 | 19 | 48 | 49 | 50 | 2, 3 |
|  | LI23R2 | 23 | 28 | 29 | 17 | 3 |
|  | LI23R3 | 23 | 29 | 30 | 9 | 3 |
| Simmental | SI13R1 | 13 | 73 | 74 | 9 | 2, 3 |
|  | SI13R2 | 13 | 74 | 75 | 15 | 2, 3 |
|  | SI13R3 | 13 | 75 | 76 | 10 | 2, 3 |
|  | SI16R4 | 16 | 51 | 52 | 9 | 1, 2 |
|  | SI16R5 | 16 | 53 | 54 | 9 | 1, 2 |
|  | SI19R6 | 19 | 48 | 49 | 12 | 2, 3 |

**Additional file 1 Table S1 Genomic regions with at least nine significant haplotypes for absence or reduced level of homozygosity at** $\boldsymbol{p<5*}\boldsymbol{10}^{\boldsymbol{-8}}$ **by breed**

1Test category: 1 – Population test, 2 – Carrier mating tests, 3 – Semi-lethality tests
