# Supplementary material for: Analysis of a large dataset reveals haplotypes carrying putatively recessive lethal and semi-lethal alleles with pleiotropic effects on economically important traits in beef cattle
Source: Genet Sel Evol. 2019 Mar 5;51:9. doi: 10.1186/s12711-019-0452-z (PMC6402105; doi:10.1186/s12711-019-0452-z)
Supplement: Supplementary file 2 — Additional file 2: Table S2. Hazard ratios [95% confidence intervals] for postnatal survival of progeny from non-carrier × carrier (HH × Hh) and carrier × carrier (Hh × Hh) matings as compared to the progeny from non-carrier × non-carrier (HH × HH) matings for the 22 identified haplotypes with significant absence or reduced level of homozygosity. The data provided represent survival analysis for different parent haplotype carrier status for several haplotypes with significant absence or reduced level of homozygosity. [file 12711_2019_452_MOESM2_ESM.docx]

**Additional file 2 Table S2** **Hazard ratios [95% confidence intervals] for postnatal survival of progeny from non-carrier × carrier (**$\boldsymbol{HH}\boldsymbol{Hh}$**) and carrier × carrier (**$\boldsymbol{Hh}\boldsymbol{Hh}$**) matings as compared to the progeny from non-carrier × non-carrier (**$\boldsymbol{HH} \boldsymbol{HH}$**) matings for the 22 identified haplotypes with significant absence or reduced level of homozygosity**

| **Haplotype** | **Censored** | |  | **Complete** | |
| --- | --- | --- | --- | --- | --- |
|  | *HH* × *Hh* | *Hh* × *Hh* |  | *HH* × *Hh* | *Hh* × *Hh* |
| AA14H1 | 1.55 _[0.98, 2.46]_ | 0.77 _[0.36, 1.65]_ |  | 0.95 _[0.77, 1.16]_ | 0.89 _[0.67, 1.17]_ |
| AA14H2 | 1.70* _[1.04, 2.80]_ | 1.70 _[0.74, 3.90]_ |  | 0.95 _[0.76, 1.19]_ | 0.92 _[0.62, 1.35]_ |
| AA14H3 | 1.34 _[0.79, 2.27]_ | 2.04 _[0.89, 4.70]_ |  | 0.86 _[0.68, 1.09]_ | 1.24 _[0.84, 1.84]_ |
| AA14H4 | 1.45 _[0.85, 2.47]_ | 1.28 _[0.49, 3.33]_ |  | 0.94 _[0.75, 1.19]_ | 0.86 _[0.58, 1.28]_ |
| AA18H5 | 1.43 _[0.89, 2.30]_ | 1.50 _[0.78, 2.85]_ |  | 0.91 _[0.74, 1.12]_ | 0.99 _[0.73, 1.34]_ |
| AA19H6 | 1.36 _[0.83, 2.22]_ | 1.74 _[0.87, 3.44]_ |  | 0.80 _[0.63, 1.01]_ | 0.99 _[0.66, 1.49]_ |
| CH13H1 | 0.88 _[0.68, 1.14]_ | 1.05 _[0.71, 1.54]_ |  | 1.00 _[0.87, 1.14]_ | 1.11 _[0.91, 1.36]_ |
| CH19H2 | 1.00 _[0.75, 1.32]_ | 1.42 _[0.86, 2.33]_ |  | 1.11 _[0.96, 1.28]_ | 1.36* _[1.05, 1.75]_ |
| CH19H3 | 0.87 _[0.64, 1.18]_ | 0.32 _[0.04, 2.26]_ |  | 0.93 _[0.80, 1.09]_ | 0.67 _[0.35, 1.29]_ |
| HE6H1 | 0.93 _[0.62, 1.40]_ | 0.79 _[0.42, 1.49]_ |  | 1.24* _[1.02, 1.50]_ | 1.01 _[0.75, 1.36]_ |
| HE6H2 | 0.84 _[0.57, 1.26]_ | 0.89 _[0.52, 1.51]_ |  | 1.17 _[0.97, 1.41]_ | 1.10 _[0.86, 1.41]_ |
| HE19H3 | 1.37 _[0.92, 2.03]_ | 1.17 _[0.58, 2.34]_ |  | 1.21 _[0.98, 1.49]_ | 1.16 _[0.73, 1.83]_ |
| LI19H1 | 1.10 _[0.87, 1.40]_ | 1.03 _[0.63, 1.68]_ |  | 0.90 _[0.80, 1.01]_ | 1.04 _[0.82, 1.31]_ |
| LI19H2 | 0.97 _[0.72, 1.31]_ | 0.66 _[0.21, 2.06]_ |  | 0.97 _[0.84, 1.11]_ | 0.63 _[0.35, 1.15]_ |
| LI23H3 | 0.94 _[0.73, 1.23]_ | 0.85 _[0.47, 1.52]_ |  | 0.92 _[0.82, 1.04]_ | 0.84 _[0.65, 1.09]_ |
| LI23H4 | 1.02 _[0.78, 1.33]_ | 1.47 _[0.86, 2.49]_ |  | 0.83* _[0.73, 0.95]_ | 0.94 _[0.71, 1.26]_ |
| SI13H1 | 0.99 _[0.53, 1.87]_ | 1.50 _[0.45, 4.94]_ |  | 0.98 _[0.75, 1.28]_ | 1.21 _[0.67, 2.17]_ |
| SI13H2 | 0.88 _[0.54, 1.46]_ | 1.60 _[0.89, 2.89]_ |  | 0.95 _[0.78, 1.17]_ | 1.30 _[0.99, 1.71]_ |
| SI13H3 | 0.84 _[0.43, 1.67]_ | 1.85 _[0.65, 5.29]_ |  | 0.94 _[0.71, 1.23]_ | 1.05 _[0.59, 1.85]_ |
| SI16H4 | 1.09 _[0.64, 1.86]_ | 1.06 _[0.32, 3.50]_ |  | 1.08 _[0.87, 1.35]_ | 1.09 _[0.67, 1.78]_ |
| SI16H5 | 1.07 _[0.59, 1.97]_ | 2.57 _[0.35,18.95]_ |  | 1.25 _[0.98, 1.60]_ | 2.36 _[0.87, 6.40]_ |
| SI19H6 | 1.06 _[0.62, 1.83]_ | 1.04 _[0.40, 2.70]_ |  | 1.02 _[0.80, 1.29]_ | 1.17 _[0.80, 1.71]_ |

*p < 0.05
