# Supplementary material for: Analysis of a large dataset reveals haplotypes carrying putatively recessive lethal and semi-lethal alleles with pleiotropic effects on economically important traits in beef cattle
Source: Genet Sel Evol. 2019 Mar 5;51:9. doi: 10.1186/s12711-019-0452-z (PMC6402105; doi:10.1186/s12711-019-0452-z)
Supplement: Supplementary file 3 — Additional file 3: Table S3. SNP alleles for the AA14H3, CH19H2, and SI16H5 haplotypes. The data provided represent the SNP alleles for haplotype (SI16H5) that carries putatively recessive lethal and two haplotypes (AA14H3, CH19H2) that carry semi-lethal alleles. [file 12711_2019_452_MOESM3_ESM.docx]

**Additional file 3 Table S3 SNP alleles for the AA14H3, CH19H2, and SI16H5 haplotypes**

| **Haplotype** | **Chromosome** | **SNP name** | **SNP position (bp)** | **SNP allele** |
| --- | --- | --- | --- | --- |
| AA14H3 | 14 | HAPMAP24518-BTC-062393 | 8064004 | C |
|  |  | HAPMAP31989-BTC-062364 | 8113083 | C |
|  |  | HAPMAP41162-BTA-115830 | 8144892 | C |
|  |  | HAPMAP27714-BTC-062128 | 8207783 | A |
|  |  | HAPMAP30140-BTC-046976 | 8241174 | A |
|  |  | ARS-BFGL-NGS-28337 | 8264685 | A |
|  |  | HAPMAP26322-BTC-062079 | 8351971 | A |
|  |  | UA-IFASA-8561 | 8385937 | A |
|  |  | HAPMAP31987-BTC-062044 | 8425401 | C |
|  |  | HAPMAP22779-BTC-061888 | 8454690 | A |
|  |  | HAPMAP26904-BTC-061747 | 8479608 | A |
|  |  | HAPMAP22964-BTC-001504 | 8529243 | G |
|  |  | HAPMAP27836-BTC-052671 | 8551460 | A |
|  |  | HAPMAP26030-BTC-052382 | 8621379 | C |
|  |  | ARS-BFGL-NGS-85289 | 8659069 | A |
|  |  | HAPMAP31945-BTC-052169 | 8698150 | C |
|  |  | HAPMAP22724-BTC-001541 | 8730305 | A |
|  |  | ARS-BFGL-NGS-19923 | 8851361 | A |
|  |  | ARS-BFGL-NGS-76248 | 8879810 | A |
|  |  | UA-IFASA-7476 | 8927881 | C |
| CH19H2 | 19 | IDBV31900008656 | 48874999 | G |
|  |  | IDBV31900008658 | 48875131 | G |
|  |  | IDBV21900002820 | 48875852 | C |
|  |  | IDBV31900008668 | 48875866 | A |
|  |  | IDBV31900008680 | 48883348 | A |
|  |  | IDBV21900002861 | 48887256 | C |
|  |  | IDBV31900008693 | 48889014 | C |
|  |  | IDBV21900002872 | 48889025 | C |
|  |  | IDBV21900002873 | 48889311 | G |
|  |  | IDBV21900002884 | 48889825 | A |
|  |  | IDBV21900002885 | 48889874 | A |
|  |  | IDBV21900002888 | 48890897 | C |
|  |  | IDBV31900008702 | 48890934 | A |
|  |  | IDBV31900008704 | 48891361 | C |
|  |  | IDBV21900002911 | 48891663 | C |
|  |  | IDBV31900008728 | 48899093 | C |
|  |  | IDBV31900008732 | 48899230 | A |
|  |  | IDBV21900002954 | 48900428 | C |
|  |  | IDBV21900002965 | 48901947 | C |
|  |  | IDBV31900008738 | 48902319 | A |
| SI16H5 | 16 | ARS-BFGL-NGS-743 | 51811400 | G |
|  |  | ARS-BFGL-NGS-173 | 51833247 | C |
|  |  | UA-IFASA-920 | 51914451 | A |
|  |  | ARS-BFGL-NGS-41367 | 51942323 | A |
|  |  | BTB-00646135 | 52141211 | A |
|  |  | BTB-00646146 | 52168565 | C |
|  |  | BTB-00646159 | 52211744 | C |
|  |  | ARS-BFGL-NGS-27516 | 52510377 | C |
|  |  | ARS-BFGL-NGS-116396 | 52786297 | A |
|  |  | ARS-BFGL-NGS-78363 | 52975189 | G |
|  |  | UA-IFASA-8741 | 53070045 | C |
|  |  | ARS-BFGL-NGS-112066 | 53097936 | G |
|  |  | ARS-BFGL-NGS-2902 | 53199787 | A |
|  |  | IDBV31600008012 | 53248415 | C |
|  |  | IDBV31600008013 | 53262642 | C |
|  |  | ARS-BFGL-NGS-4009 | 53279306 | A |
|  |  | BTA-39204-NO-RS | 53330998 | G |
|  |  | ARS-BFGL-NGS-8174 | 53354164 | C |
|  |  | IDBV31600008014 | 53419817 | G |
|  |  | ARS-BFGL-NGS-117470 | 53434159 | A |
