# Supplementary material for: Analysis of a large dataset reveals haplotypes carrying putatively recessive lethal and semi-lethal alleles with pleiotropic effects on economically important traits in beef cattle
Source: Genet Sel Evol. 2019 Mar 5;51:9. doi: 10.1186/s12711-019-0452-z (PMC6402105; doi:10.1186/s12711-019-0452-z)
Supplement: Supplementary file 4 — Additional file 4: Table S4. Estimated economic effect for the AA14H3, CH19H2, and SI16H5 haplotypes. The data provided represent estimated economic impact for haplotype (SI16H5) that carries putatively recessive lethal and two haplotypes (AA14H3, CH19H2) that carry semi-lethal alleles. [file 12711_2019_452_MOESM4_ESM.docx]

**Additional file 4 Table S4 Estimated economic effect for the AA14H3, CH19H2, and SI16H5 haplotypes**

| **Haplotype** | **AA14H3** | **CH19H2** | **SI16H5** |
| --- | --- | --- | --- |
| Number of calves with purebred sire and purebred dam | 9898 | 12,100 | 4306 |
| Number of calves with purebred sire and crossbred dam | 136,081 | 218,893 | 170,648 |
| Number of calves with crossbred sire and crossbred dam | 19,277 | 16,230 | 9037 |
| Haplotype frequency | 0.152 | 0.144 | 0.088 |
| Insemination success rate | 0.520 | 0.423 | 0.452 |
| Number of days between two inseminations | 70 | 69 | 60 |
| Haplotype substitution effect for terminal index | 3.424 | 1.469 | 2.299 |
| Costs of keeping cow in herd (€) | 2.2 | 2.2 | 2.2 |
| Number of homozygous embryos | 3677 | 6157 | 1574 |
| Economic loss due to the lethality (€) | 566,257 | 934,673 | 207,810 |
| Number of heterozygous calves born | 33,141 | 47,894 | 22,655 |
| Economic gain for terminal index (€) | 113,475 | 70,356 | 52,084 |
| Net effect (€) | -452,782 | -864,317 | -155,726 |
