# Supplementary figures and images for: Analysis of a large dataset reveals haplotypes carrying putatively recessive lethal and semi-lethal alleles with pleiotropic effects on economically important traits in beef cattle
Source: Genet Sel Evol. 2019 Mar 5;51:9. doi: 10.1186/s12711-019-0452-z (PMC6402105; doi:10.1186/s12711-019-0452-z)

Net effect (EUR)

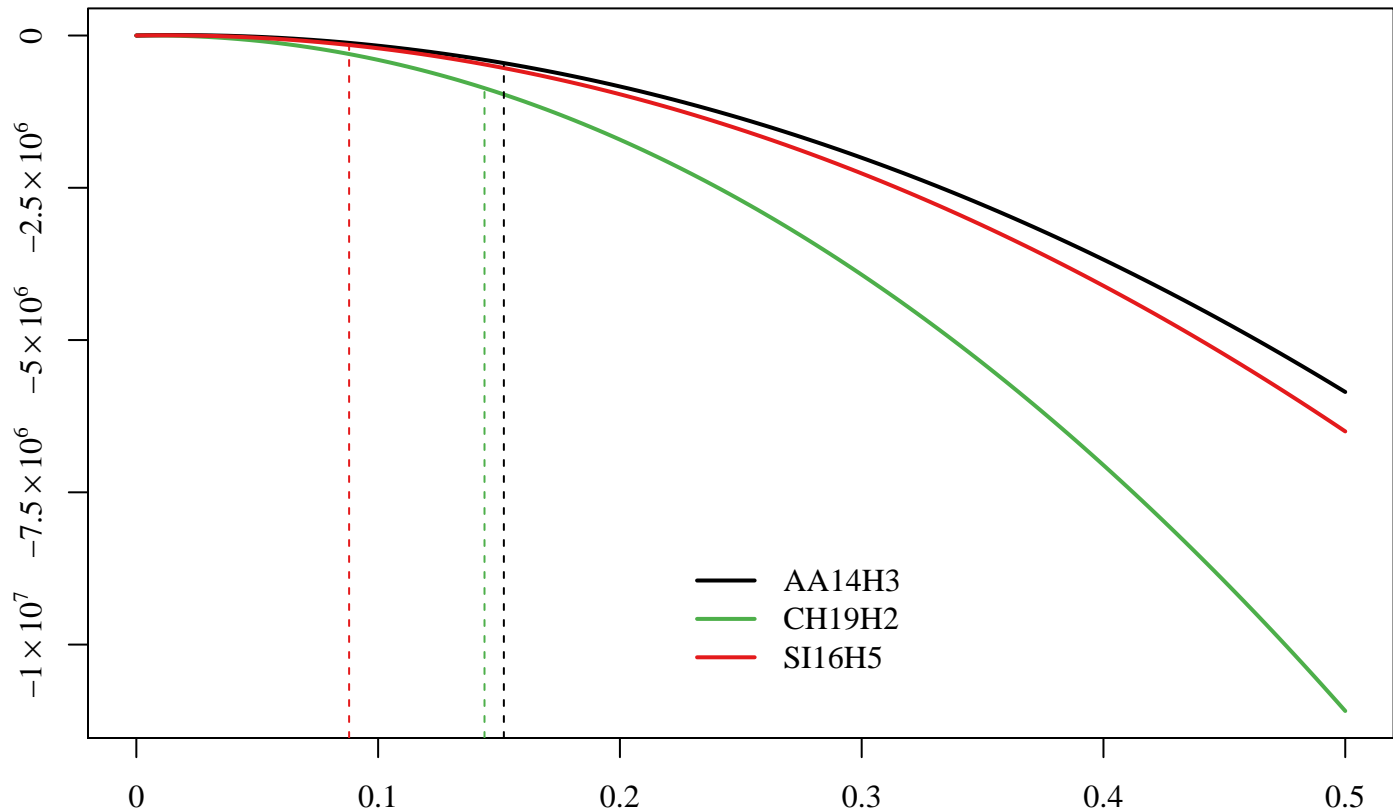

Haplotype frequency

Supplement: Supplementary file 5 — Additional file 5: Figure S1. Estimated national annual net effect for the AA14H3, CH19H2, and SI16H5 haplotypes with vertical lines showing the net effect under the current haplotype frequencies. The figure provided shows the net effect for haplotype (SI16H5) that carries putatively recessive lethal and two haplotypes (AA14H3, CH19H2) that carry semi-lethal alleles for different haplotype frequencies. [file 12711_2019_452_MOESM5_ESM.pdf]
