# Supplementary material for: Analysis of a large dataset reveals haplotypes carrying putatively recessive lethal and semi-lethal alleles with pleiotropic effects on economically important traits in beef cattle
Source: Genet Sel Evol. 2019 Mar 5;51:9. doi: 10.1186/s12711-019-0452-z (PMC6402105; doi:10.1186/s12711-019-0452-z)
Supplement: Supplementary file 6 — Additional file 6: Table S5. Protein coding genes between 51,611,400 and 53,234,159 bp on bovine chromosome 16 for the SI16H5 haplotype; the genes showing prenatal or perinatal lethality in mice are in bold. The data provided represent protein coding genes located on the haplotype (SI16H5) that carries putatively recessive lethal allele. [file 12711_2019_452_MOESM6_ESM.docx]

**Additional file 6 Table S5 Protein coding genes between 51,611,400 and 53,234,159 bp on bovine chromosome 16 for the SI16H5 haplotype; the genes showing prenatal or perinatal lethality in mice are in bold**

| **Ensembl gene id** | **Start base** | **End base** |
| --- | --- | --- |
| **ENSBTAG00000038716** | **51,766,102** | **51,819,356** |
| ENSBTAG00000014136 | 51,841,819 | 51,846,448 |
| ENSBTAG00000014119 | 51,848,098 | 51,947,503 |
| ENSBTAG00000008672 | 51,963,930 | 51,974,480 |
| ENSBTAG00000005231 | 52,023,631 | 52,076,607 |
| ENSBTAG00000024510 | 52,078,217 | 52,081,782 |
| ENSBTAG00000024509 | 52,083,968 | 52,085,908 |
| ENSBTAG00000024508 | 52,086,276 | 52,086,936 |
| **ENSBTAG00000000215** | **52,106,960** | **52,184,132** |
| ENSBTAG00000000212 | 52,203,712 | 52,212,869 |
| ENSBTAG00000031749 | 52,222,594 | 52,236,396 |
| ENSBTAG00000010737 | 52,240,501 | 52,260,332 |
| ENSBTAG00000039728 | 52,249,583 | 52,372,101 |
| ENSBTAG00000010732 | 52,260,743 | 52,263,073 |
| ENSBTAG00000010720 | 52,264,771 | 52,276,754 |
| ENSBTAG00000047755 | 52,297,147 | 52,298,167 |
| ENSBTAG00000002575 | 52,310,820 | 52,325,725 |
| ENSBTAG00000046073 | 52,330,676 | 52,336,051 |
| **ENSBTAG00000002219** | **52,336,171** | **52,352,541** |
| ENSBTAG00000021294 | 52,360,276 | 52,365,505 |
| ENSBTAG00000023784 | 52,370,233 | 52,373,541 |
| ENSBTAG00000039055 | 52,376,216 | 52,379,152 |
| ENSBTAG00000004872 | 52,383,424 | 52,386,462 |
| ENSBTAG00000004873 | 52,388,692 | 52,396,825 |
| ENSBTAG00000004874 | 52,398,515 | 52,402,983 |
| ENSBTAG00000012247 | 52,424,102 | 52,428,456 |
| ENSBTAG00000010696 | 52,431,328 | 52,440,427 |
| ENSBTAG00000015882 | 52,441,615 | 52,449,785 |
| ENSBTAG00000031631 | 52,452,723 | 52,456,578 |
| ENSBTAG00000037523 | 52,467,804 | 52,468,793 |
| ENSBTAG00000015642 | 52,474,085 | 52,481,382 |
| ENSBTAG00000015635 | 52,484,468 | 52,487,309 |
| ENSBTAG00000015632 | 52,492,065 | 52,494,746 |
| ENSBTAG00000018028 | 52,499,838 | 52,514,414 |
| ENSBTAG00000007623 | 52,572,639 | 52,576,496 |
| ENSBTAG00000007620 | 52,576,620 | 52,586,919 |
| ENSBTAG00000007619 | 52,586,830 | 52,589,663 |
| ENSBTAG00000017674 | 52,595,746 | 52,599,094 |
| ENSBTAG00000009856 | 52,601,203 | 52,610,521 |
| ENSBTAG00000014511 | 52,642,150 | 52,651,239 |
| ENSBTAG00000046758 | 52,656,951 | 52,657,700 |
| ENSBTAG00000024492 | 52,660,395 | 52,665,705 |
| ENSBTAG00000013191 | 52,674,207 | 52,712,338 |
| ENSBTAG00000014707 | 52,714,627 | 52,715,665 |
| ENSBTAG00000014705 | 52,724,734 | 52,725,748 |
| ENSBTAG00000014540 | 52,742,637 | 52,748,240 |
| ENSBTAG00000014537 | 52,748,704 | 52,755,937 |
| ENSBTAG00000013813 | 52,756,766 | 52,762,408 |
| ENSBTAG00000016528 | 52,763,919 | 52,775,777 |
| ENSBTAG00000008739 | 52,776,143 | 52,794,718 |
| **ENSBTAG00000018730** | **52,852,315** | **52,881,022** |
| ENSBTAG00000018729 | 52,883,152 | 52,974,692 |
| ENSBTAG00000018725 | 53,027,481 | 53,049,143 |
| ENSBTAG00000018724 | 53,064,138 | 53,068,821 |
| ENSBTAG00000018723 | 53,069,568 | 53,074,055 |
| ENSBTAG00000018722 | 53,075,569 | 53,115,375 |
| ENSBTAG00000039662 | 53,131,205 | 53,133,055 |
| ENSBTAG00000018718 | 53,135,568 | 53,168,210 |
| ENSBTAG00000002478 | 53,199,433 | 53,208,249 |
| ENSBTAG00000002476 | 53,208,896 | 53,248,680 |
| **ENSBTAG00000002472** | **53,250,584** | **53,273,042** |
| ENSBTAG00000024496 | 53,272,046 | 53,288,451 |
| ENSBTAG00000024497 | 53,295,217 | 53,302,109 |
| ENSBTAG00000009048 | 53,306,776 | 53,324,889 |
| ENSBTAG00000002408 | 53,336,075 | 53,469,243 |
